# Gender-based differences in cardiac diseases

**DOI:** 10.1016/S1674-8301(11)60010-9

**Published:** 2011-03

**Authors:** Pei-Chi Yang, Colleen E. Clancy

**Affiliations:** Department of Pharmacology, University of California Davis. Davis, CA 96516-5270, USA.

**Keywords:** gender differences, heart failure, Brugada syndrome, long QT

## Abstract

It has been observed that the incidence of heart failure and Brugada syndrome are higher in men, while women are more likely to have QT interval prolongation and develop torsades de pointes (TdP). Over the past decade, new studies have improved our understanding of the mechanisms of abnormal repolarization and the relationship between gender differences in cardiac repolarization and presentation of clinical syndromes. Nevertheless, the causes of gender-based differences in cardiac disease are still not completely clear. This review paper briefly summarized what is currently known about gender differences in heart failure, Brugada syndrome and long QT syndrome from molecular mechanisms to clinical presentations.

## INTRODUCTION

In the past decade, it has become increasingly clear that cardiac arrhythmia and heart failure (HF) have gender-based differences that increase or reduce disease susceptibility[1]–[3]. The mechanisms of arrhythmia initiation, sustenance and termination as well as HF presentation appear to be gender specific. Recent clinical and experimental studies suggest that these differences may stem in part from fundamental intrinsic gender differences in cardiac tissue[4]–[10]. These include intrinsic electrical differences resulting from variable ion channel expression and diverse sex hormonal regulations via long-term genomic and acute nongenomic pathways[6],[11]–[14], though the exact role gender plays in cardiac diseases is not fully understood.

## GENDER-RELATED DIFFERENCES IN ELECTROPHYSIOLOGICAL REMODELING WITH HEART FAILURE

In HF, the heart cannot supply an adequate amount of blood to the rest of body. Blood moves to the heart and body at a slower rate, and pressure increases in the heart. In order to sustain cardiac performance, the chambers of the heart stretch to hold more blood to pump through the body by becoming thickened and stiff. For a short period of time, this helps to maintain the blood pressure, but eventually leads to cardiac dysfunction[15],[16].

The common causes of HF include ischemic heart disease, cigarette smoking, hypertension, obesity, diabetes mellitus, and valvular heart disease. The causes of HF are difficult to analyze because of differences in gender, race and prevalence of causes changing with age. Clinical data confirm that HF is more common in patients older than 50 years[17] when testosterone levels are reduced. A number of studies have also found low levels of testosterone in HF patients[18], and have shown measurable short-term benefits from testosterone therapy[19],[20]. However, no clear predictive role of testosterone levels has been defined. In addition, clinical trials have shown that the progression of HF is slower in women than in men, and females have improved survival in HF[21]–[25]. Compared to men, women tend to develop HF at older ages[26]. Interestingly, women are more likely to develop diastolic HF with normal left ventricular ejection fraction compared with men[39]–[41].

Thus, although sex differences have been observed in HF, the underlying mechanisms are still not clear. There are a number of recent detailed reviews on ion-channel remodeling in HF[27]–[30] and gender differences in quality of life in HF patients[26],[31],[32]. Here, we focus on gender differences in some of the major channels and transporters during electrophysiological remodeling.

It is well known that HF causes cardiac functional changes. These changes make the heart prone to arrhythmias and diastolic and systolic contractile dysfunction. One of the important regulators of cardiac contractile function is phospholamban (PLB). During systole, PLB binds to a Ca^2+^ pump and prevents Ca^2+^ from being pumped back into the sarcoplasmic reticulum (SR). During muscle relaxation, PLB is in its phosphorylated state, which removes its inhibitory effect on the SR Ca^2+^-ATPase (SERCA) and restores low calcium levels in the cytoplasm[33]. In a gene expression study, PLB is found highly expressed in human failing hearts[34], and may be a mechanism of systolic contractile dysfunction[35]. Notably, in men, the expression levels of PLB are increased[36]. PLB has also been shown to be phosphorylated by cAMP-dependent protein kinase and Ca^2+^/calmodulin-dependent protein kinase[37],[38]. Calmodulin-3 has a lower expression level in men[36].

The activity of the Na^+^/K^+^-ATPase via its interaction with the Na^+^/Ca^2+^ exchanger (NCX) is important for maintaining Ca^2+^ homeostasis in the heart. HF studies have found reduced expression of Na^+^/K^+^-ATPase-α1 in human failing heart tissue[34],[42]. This may lead to decreasing Ca^2+^ efflux by NCX, which increases cytoplasmic Ca^2+^ concentration and causes development of Ca^2+^-dependent arrhythmias. In a gender difference study, it was discovered that men had reduced expression-levels of Na^+^/K^+^-ATPase-α1[36]. In addition, the plasma membrane Ca^2+^-ATPase isoform 4 was found to be less strongly expressed in HF mice[43] and in men[36].

Other cardiac functional changes in HF include action potential duration (APD) prolongation, reduction of cell excitability[44], increased Na^+^/Ca^2+^ exchange, preserved β-adrenergic responsiveness, and reduced outward K^+^ currents (I_to_ or I_K1_), which may contribute to APD prolongation[45]. A HF study in porcine myocytes demonstrated that NCX is more phosphorylated in male pacing-induced failing swine and that β-adrenergic responsiveness was greatly reduced in males compared to females[46]. This study suggested that increased NCX activity could lead to impaired contractile function by decreasing SR Ca^2+^ content and promote the development of arrhythmia triggers. Females may have better survival rates in HF because they have a smaller NCX current and larger preserved β-adrenergic regulation.

In ischemic myocytes, high levels of intracellular Na^+^ cause membrane potential changes that enhance Ca^2+^ influx via NCX. This increased influx could lead to Ca^2+^ “overload”. Various studies have been conducted to investigate the female gender in cardio-protection during ischemia and suggest a protective role of estrogen in hypertrophied and/or failing myocardium[47]–[51]. One recent study showed that acute effects of estrogen at physiological concentration (1 nmol/L) reduced the increase in [Na^+^]_i_ during metabolic inhibition (MI), and suggested that estrogen may regulate Ca^2+^ influx through reverse NCX by lessening the magnitude of the rise in [Na^+^]_i_ during MI in ischemic hearts[47].

## BRUGADA SYNDROME AND MEN

East Asia is an area of high prevalence of Brugada syndrome (BrS), and the male-female ratio of the clinical phenotype is 8:1[52],[53]. BrS is a polymorphic ventricular tachycardia characterized by ST-segment elevation in the right precordial leads (V_1_–V_3_) and right bundle branch block. BrS has been linked to a number of mutations in the gene *SCN5A* encoding the cardiac Na^+^ channel, all of which cause loss of channel function[54]–[60].

Two hypotheses have been discussed recently — the “repolarization hypothesis” and “depolarization hypothesis”. The repolarization hypothesis is based on evidence for transmural dispersion of repolarization between the canine right ventricle (RV) epicardium and endocardium[61]. Early repolarization due to loss of the AP-dome in the epicardium is expected to occur, which may induce phase 2-reentry and a substrate for the development of VT/VF[5],[62],[63].

On the other hand, the depolarization hypothesis proposed by Wild and Postema suggests RV conduction delay as part of the pathophysiological mechanism of BrS that is supported by clinical data[64]. They propose that depolarization abnormalities with mild structural abnormalities[65] may explain the clinical observed repolarization abnormalities on the electrocardiogram (ECG). Antzelevitch *et al*[63]. agree that slowed conduction and mild structural defects exist in some BrS cases, especially in I_Na_ loss of function cases, but it is not absolutely required. They pointed out that noticeable accentuation of the epicardial AP notch can account for the ST segment elevation associated with BrS by causing loss of the AP dome in some cells and not others, leading to a dispersion of repolarization. Dispersion of repolarization might allow a premature beat to trigger reentrant arrhythmias[65].

**Fig. 1:**
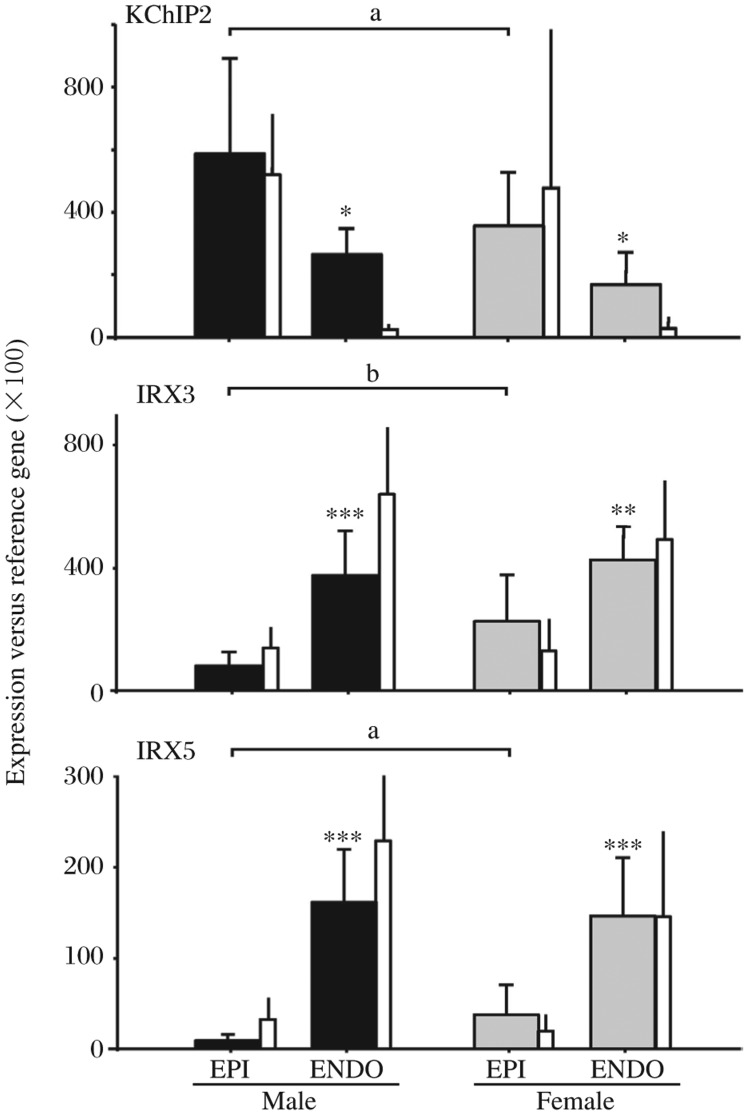
Expression-profile of KChIP2 and Iroquois transcription factors. Thick bars show transcript-expression in the right ventricle (RV). The left ventricle (LV)-data are represented by narrow bars. I_to_ β-subunit KChIP2 was expressed at higher levels in the RV-EPI, and was higher in males than females. No gender difference in epicardial KChIP2-expression was found in the LV. In human hearts, IRX3 and IRX5 (the Iroquois transcription factors) control expression of transient outward K^+^ currents and were strongly expressed in the ENDO *vs* EPI, an inverse pattern to KChIP2. Moreover, in the RV-EPI, IRX3 and IRX5 were expressed at higher levels in females *vs* males (Thick bars). On the other hand, the expression-levels of these two genes were lower in females than in males in the LV-ENDO (narrow bars). *P < 0.05, **P < 0.01, ***P < 0.001 *vs* EPI. a: *P* < 0.05, b: *P* < 0.01 *vs* women. LV ENDO *vs* EPI, Male: KChIP2, *P* < 0.001; IRX3, *P* < 0.001; IRX5, *P* < 0.001; Female: KChIP2, *P* < 0.01; IRX3, *P* < 0.001; IRX5, *P* < 0.01. EPI: epicardium; ENDO: endocardium. Reprinted with permission from Gaborit *et al*. (2010) [36] with modification.

An ionic and cellular basis for the intriguing sex-related distinction in presentation of BrS was first proposed by Di Diego and colleagues[4]. They suggested that a more prominent I_to_ in males leads to the predominance of Brugada phenotype in men. In a recent ion-channel expression-pattern study, Gaborit *et al*. found lower-level expression of repolarizing ion-channel subunits including KCHIP2, HERG, Kir2.3, Kir6.2 and SUR2 in females that may protect them against the Brugada phenotype (but also make them susceptible to long QT as discussed below). In male RV-epicardium, higher-level expression of the I_to_ β-subunit KChIP2 has been observed. Larger male current may favor early repolarization in Brugada-patients[36]. However, no gender-differences were found in epicardial KChIP2-expression in human LV (***Fig. 1***). This finding is compatible with a canine model of sex-related differences conducted by Di Diego *et al*.[4], showing no sex differences on I_to_ current and epicardial phase-1 repolarization in LV.

## LONG QT SYNDROME AND WOMEN

**Fig. 2:**
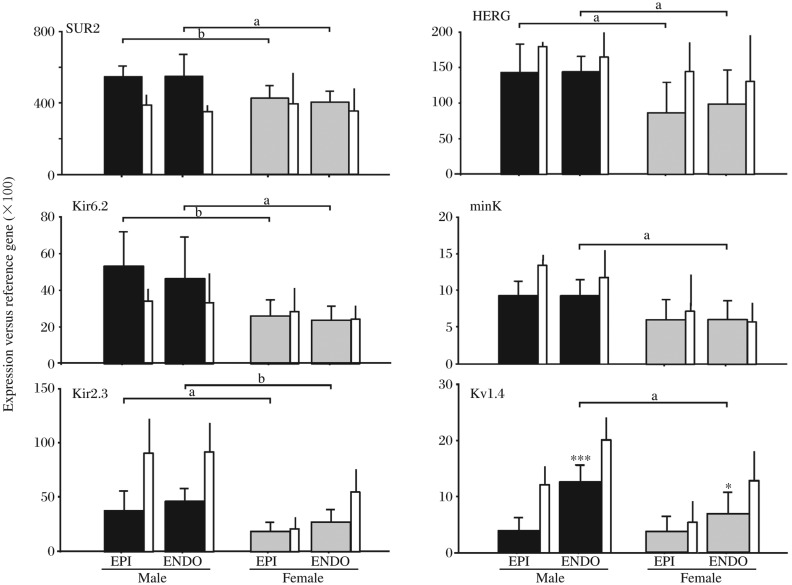
Expression-profile of gender-differential K^+^-channel genes. The same format as in *Fig. 1*. The results are expressed as mean±SD from 7 donors/gender. In both the EPI and ENDO, the inward-rectifier Kir6.2 and Kir2.3 were weakly expressed in women. The regulator of Kir6.2, SUR2, was lower in female right ventricle (RV). The rapid delayed-rectifier HERG was strongly expressed in male hearts in both the EPI and ENDO. In female, the I_Ks_ β-subunit minK was expressed at lower levels, and Kv1.4 was strongly expressed in male ENDO. *P < 0.05, ***P < 0.001 *vs* EPI. a: *P* < 0.05, b: *P* < 0.01 *vs* women. LV-statistics: EPI *vs* ENDO: male, Kv1.4, *P* < 0.01; female, Kv1.4, *P* < 0.05, Kir2.3, *P* < 0.01. Male EPI *vs* Female: Kir2.3, *P* < 0.001; HERG, *P* < 0.05; minK, *P* < 0.01; Kv1.4, *P* < 0.01. Male ENDO *vs* Female: Kir2.3, *P* < 0.05; minK, *P* < 0.01; Kv1.4, *P* < 0.05. EPI: epicardium; ENDO: endocardium. Reprinted with permission from Gaborit *et al*. (2010) [36] with modification.

Female gender is a determinant of susceptibility to certain types of cardiac arrhythmia. For example, female sex is a risk factor for inherited and acquired long-QT (LQT) syndrome and associated with torsade de pointes (TdP) arrhythmias[9],[12],[66]–[69]. Various studies have shown that females have a higher risk of a first cardiac event between 15 and 40 years[70], and observed that women are at higher risk than men of drug-induced TdP by class III anti-arrhythmic drugs and other drugs that block HERG[71]–[78]. Animal studies have shown higher-level inward currents in females[67],[79],[80]. These agree with a recent expression-pattern study, where the authors found lower expression-levels of K^+^ channel α- (Kir2.3, Kv1.4 and HERG) and β- (minK) subunits in female heart[36]. The differences between male and female HERG were significant in RV only, while the sex differences on Kir2.3, Kv1.4 and minK were significant in both RV and LV (***Fig. 2***)[36].

The fact that women are at particular risk for drug-induced arrhythmias and that arrhythmia risk rises around the time of puberty, suggests the dominant female hormones estrogen and progesterone modulate arrhythmia vulnerability. While estrogen may exacerbate arrhythmia susceptibility[8],[81] by directly interacting with the drug binding site on the promiscuous hERG subunit and reducing I_Kr_ current and increasing the rate of channel deactivation[81], progesterone is apparently protective and reduces QT intervals[82]. Studies suggested that both progesterone and testosterone acutely modulate I_Ks_ and I_CaL_ through phosphoinositide 3-kinase (PI3K)/AKT-dependent endothelial nitric oxide (NO) synthase (eNOS) activation pathway[11],[82], resulting in suppressing I_CaL_ currents and increasing I_Ks_ current density.

It has been recently suggested that the N-terminal truncated isoform of the androgen receptor (AR45) plays an essential role in the heart since the transcript level of the AR45 is high in human heart tissue. An experiment of AR45 effects on the HERG potassium channel demonstrated that AR45 enhanced HERG channels by stabilizing HERG channel protein via ERK1/2 stimulations[83]. Other studies also indicated that the male hormone testosterone (5a-DHT) increased repolarizing K^+^ currents density (I_K1_ and I_Kr_) and acts to protect against arrhythmia initiation[11],[74],[78],[84].

Nakagawa *et al*.[85] have observed that during the follicular phase (prior to ovulation) of the menstrual cycle, QT interval is longer than that in the luteal phase (following ovulation) when progesterone is increased. Arrhythmic events associated with acquired and inherited LQTs are significantly reduced during phases where progesterone level is high[85]. Moreover, QT is significantly increased by estrogen hormone replacement therapy in females and susceptibility to drug-induced arrhythmias is exaggerated in the late follicular phase where estrogen level is the highest[85]. In contrast, Burke *et al*.[86] found that in pre-menopausal women the corrected QT (QTc) interval does not greatly change through the menstrual cycle, but QTc is reduced in the luteal phase after autonomic blockade. Furthermore, one study showed that QTc did not change during the menstrual cycle, but its shortening was more pronounced in the luteal phase with ibutilide application in women[87]. The disparity in these studies may be due to the fact that corrected QT interval measurements were based on a single point or a few points with the individual patient at rest. Such an analysis is unlikely to be sensitive enough to observe significant individual differences in QT intervals as they fluctuate throughout the menstrual cycle since biological variability between patients may be larger than fluctuations in individual patients.

In addition, some drug studies demonstrated that females have greatly increased QT intervals compared with males during treatment with d,I-sotalol[73] and quinidine[88]–[90]. I_Kr_ blockers seem to increase early after depolarization (EAD) development and prolong repolarization in females, both primary and critical predictors of drug-induced TdP[91],[92].

## SIMULATION APPROACH TO UNDERSTAND EFFECTS OF SEX STEROID HORMONES AND DRUGS

**Fig. 3:**
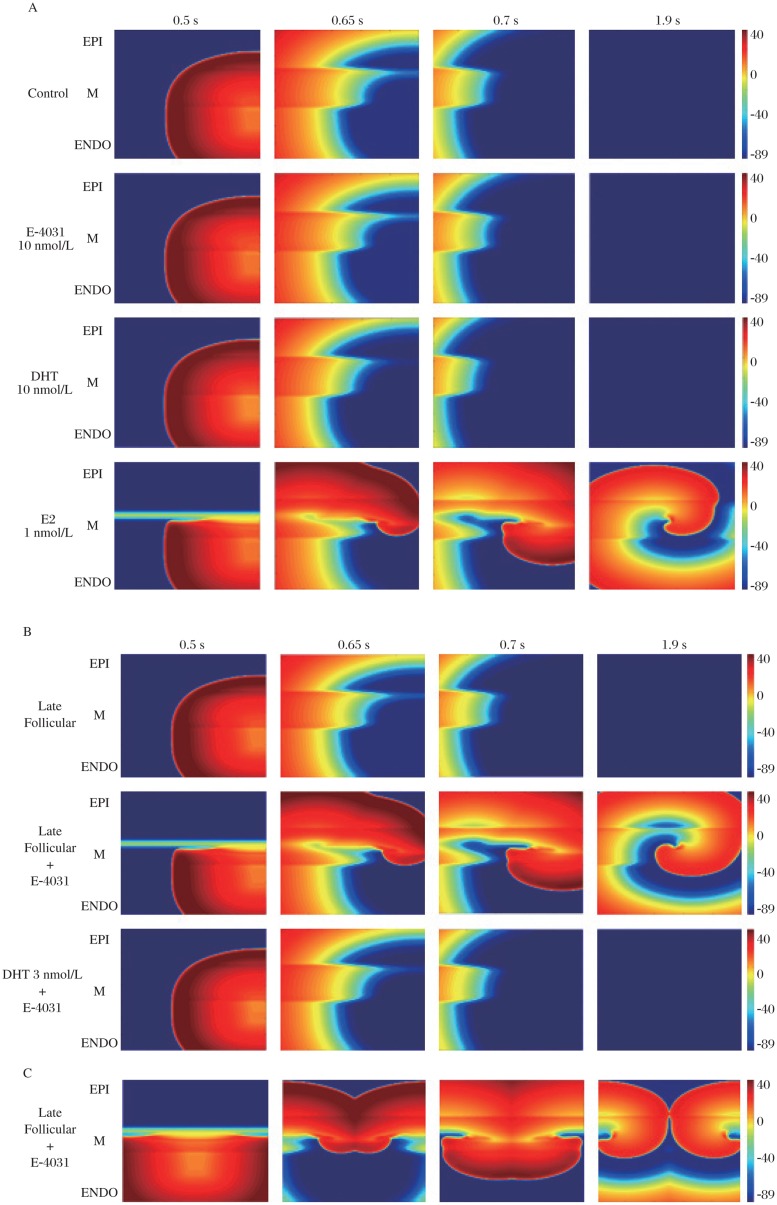
2D heterogeneous tissue simulations during short-long-short pacing protocols. Four snapshots following application of hormones and/or drug at indicated time points. Tissues were stimulated along one edge and propagated from the endocardial to the epicardial region followed by a point stimulus applied in the right edge of the endocardial region. Voltages are indicated by color gradient. A: In the absence of hormones or drugs, no reentry occurs (first row). The same behavior is observed following drug application alone (E-4031) and with testosterone application alone (DHT 10 nmol/L). However, when estrogen (E2) (1 nmol/L) is present (bottom row), the reentry was induced. B: Comparison of 2D heterogeneous tissue dynamics in the absence or presence of E-4031 during the late follicular phase, and application of testosterone 3 nmol/L with E-4031 addition. The simulation results show no reentrant activity during the late follicular phase of the menstrual cycle (progesterone, 2.5 nmol/L and E2, 1 nmol/L). However, during the late follicular phase, a spiral wave is readily induced when 10 nmol/L E-4031 is applied. Finally, testosterone 3 nmol/L with 10 nmol/L E-4031 did not trigger reentry activity. C: The same protocol as above was used, but the premature stimulus was applied during the vulnerable window in the middle of the endocardial tissue near the boundary between the endocardial region and M cells. The late follicular phase with E-4031 is shown. Reentry was introduced in this condition. The results show the effect of a point stimulus applied in the middle of the endocardial tissue, leading to the initiation of a pair of counter-rotating spiral waves. EPI: picardium; ENDO: endocardium. Reproduced with permission from Yang *et al*. (2010)[93] with modification.

It is challenging to determine the role of gender experimentally in complex cardiac functioning since gender effects are multi-factorial and affect cardiac components at different scales of the cardiac system. However, a computational approach can be useful in this respect as it allows study of specific effects in isolation without other perturbations to the system. For example, it is not easy to determine how much a role physiological concentrations of circulating sex steroid hormones play in gender linked arrhythmia susceptibility. Computational models can incorporate the effects of sex hormones measured experimentally and test these changes specifically from non-linear interactions within cells, between cells and among various tissue components that culminate to produce the overall effects of gender on the heart. In this case, simulations can be used to investigate how acute sex hormones and drugs affect system behavior[93]. The tissue simulations shown in ***Fig. 3*** predict the effects of sex steroid hormones on clinically observed QT intervals and on drug-induced LQTS. Estrogen significantly increases susceptibility to drug-induced arrhythmias. However, low concentrations of testosterone are sufficient to protect against drug-induced arrhythmias (***Fig. 3***). Our simulation studies have resulted in improved understanding of mechanisms of estrogen-mediated susceptibility to drug-induced arrhythmia initiation[93] and protective effects of progesterone and testosterone against congenital and drug-induced LQT syndrome[82],[93]. Moreover, theoretical studies have revealed gender effects at the cellular and tissue-levels as well as predicted effects of sex steroid hormone on the body surface by computing “pseudo” electrocardiograms[82],[93].
